# Formation and Evaluation of an Academic Elective for Residents in a Combined Internal Medicine-Pediatrics Residency Program

**DOI:** 10.7759/cureus.16287

**Published:** 2021-07-09

**Authors:** Benjamin M Drum, Clinton R Sheffield, John Mulcaire-Jones, Casey Gradick

**Affiliations:** 1 Internal Medicine-Pediatrics, University of Utah School of Medicine, Salt Lake City, USA; 2 Pediatric Critical Care, University of Utah School of Medicine, Salt Lake City, USA

**Keywords:** wellness, anti-racism, resident as teacher, academic month, curriculum, resident education, medical education, evidence-based clinical practice

## Abstract

Background

Recently, there has been increasing focus on skills that are crucial for success in residency that is not explicitly taught. Specifically, the four domains of teaching skills, evidence appraisal, wellness, and education on structural racism have been identified as topics that are important and underrepresented in current resident education curriculums, largely due to time constraints.

Methods

A task force consisting of one post-graduate year 2 (PGY-2) resident, one PGY-4 resident, the Associate Program Director, and the Program Director of the Internal Medicine-Pediatrics residency program was formed to explore current deficiencies in resident curriculum and to research possible solutions. As an intervention, we created and executed a four-week academic elective with dedicated time for upper-level residents to learn and explore the four domains of resident teaching, evidence-based clinical practice, wellness, and anti-racism work. The elective included several clinical sessions dedicated to implementing the skills taught in the elective. The month-long elective completed in January 2021. All residents evaluated each lecture or experience based on how valuable it was to their education on a Likert scale from 1 to 7, with 1 defined as “not valuable at all” and 7 defined as “extremely valuable.”

Results

Residents rated the overall value of teaching in each domain highly. Education and activities in wellness lectures were found to have the highest value-added material (6.20 ± 0.41, n = 18), followed by residents-as-teachers lectures (5.93 ± 0.25, n = 48), anti-racism (5.57 ± 1.11, n = 9), and evidence-based clinical practice (5.18 ± 0.50*, *n = 43). In addition, each domain was found to have at least one high-yield topic.

Conclusions

We were able to create and execute an academic elective with dedicated time for upper-level residents to develop and utilize valuable skills in teaching, evidence appraisal, wellness, and anti-racism. Future work will focus on refining the curriculum based on resident evaluations and expanding this elective to the Internal Medicine and Pediatrics categorical programs at our institution.

## Introduction

Medical residency is a complex educational process, where a resident experiences graduated autonomy over the course of several years with the goal of mastering six domains defined as core competencies by the Accreditation Council on Graduate Medical Education (ACGME): medical knowledge, interpersonal and communication skills, professionalism, systems-based practice, patient care and procedural skills, and practice-based learning and improvement [1]. While there are several core rotation requirements designed to standardize education, there is also an elective time where the resident is able to customize educational experiences to their future career. Recently, there has been increasing focus on skills that are crucial for success in residency that is not explicitly taught, including teaching skills, evidence appraisal, wellness, and education on structural racism. Although these domains have been accepted as important, programs have struggled to incorporate curricula explicitly addressing these concepts in residency [2-5].

Although regulatory bodies including the ACGME and the Liaison Committee on Medical Education require that residents assume teaching roles, there is limited preparation for teaching in medical school and no standardized curriculum in residency, with many programs not offering Residents as Teachers (RaT) curriculum [6]. As a result, many residents report feeling unprepared for an academic career [7]. A recent review of RaT curricula found that a variety of teaching methods, clinical supervision, and educational leadership skills were important components of RaT instruction and suggested that focusing on communication skills, giving feedback, and role modeling was important for a successful curriculum [8]. In addition, a review of RaT curricula found that targeted teaching can significantly improve resident teaching skills across a variety of disciplines including pediatrics and internal medicine [9].

Likewise, attaining proficiency in evidence-based clinical practice (EBCP) is an ACGME requirement, with residents expected to be able to locate, appraise, and use scientific evidence to make clinical decisions [10]. Although numerous residency programs provide education by way of journal clubs, few have a formalized curriculum to teach EBCP. A survey of internal medicine residency programs found that only one-third of programs have freestanding EBCP curricula, and less than half of those curricula had medical information resources [11]. A review of emergency medicine residents found that most residents have only moderate ability in critical appraisal [12]. There are significant barriers to residents' practicing evidence-based medicine including limited available time, attitude, and knowledge and skills [13]. Furthermore, a more recent review of EBCP education in family medicine residency programs emphasized the need for increased time for EBCP based education [5].

Recently, there has been a shift to prioritize resident wellness, as several studies have shown burnout rates as high as 55%-76% in internal medicine residency programs [14]. Barriers to resident wellness include long hours, inconsistent work environments with changing roles and identity, exposure to trauma, and lack of wellness initiatives and time to reflect [15]. Encouragingly, wellness curricula including mindfulness have been shown to increase resiliency [16]. However, research on wellness curricula is sparse, with some studies demonstrating that curricula implementation has been limited by lack of time despite showing possible efficacy [17].

Lastly, in response to a bevvy of research showing structural racism exists in the American medical system and contributes to health care inequality, there has been a push to explicitly educate residents on the role of race in medicine [18,19]. There is increasing evidence that doctors must have a role in combating racism in order to improve health care outcomes [20]. Material has mostly been developed at the level of medical student education, including curricula on the medical student level aiming to increase cultural competency, with one program explicitly using art as a medium for initiating conversations regarding structural inequality [21]. There is very limited research on residency-based anti-racism curricula.

In response to potential educational gaps in these four domains of RaT, EBCP, wellness, and anti-racism, we created a four-week academic elective where the upper-level residents of the University of Utah Internal Medicine-Pediatrics (Med-Peds) program were given dedicated time to learn and practice skills in each domain. Clinically integrated teaching of evidence-based medicine, in particular, has been shown to improve skills, attitudes, and behaviors compared to standalone teaching [22]. In addition, multiple educators have demonstrated the benefit to adult learners with just-in-time learning and on-the-job training [23,24]. With this in mind, the elective also included several opportunities for residents to implement and teach back learned skills in clinical settings during the “Core Practice” sessions.

## Materials and methods

Formation and development

A task force consisting of one second-year resident, one fourth-year resident, the Associate Program Director, and the Program Director was formed in early 2020 to explore current deficiencies in resident career formation and to research interventions. The task force surveyed our residency program and performed a literature review to identify similar academic electives at peer institutions, which were then used as a foundation for the elective.

The upper-level (third- and fourth-year) residents of the Med-Peds program (n = 5) were scheduled to have a combined four-week elective together during the 2020-2021 year. These residents were surveyed to determine which topics would be of most value.

The two residents of the task force were given a one-week elective in April 2020 to develop the curriculum and plan the tentative schedule. During this week, they combined the results of the literature review and the resident survey to identify the most salient topics to include in the elective, and four common themes emerged: RaT, EBCP, wellness, and anti-racism teaching. They met with several stakeholders including faculty members with a special interest and expertise in the common themes identified. At the end of this week, key documents including the goals and objectives of the course, course requirements, a key resources document, and a draft schedule were completed (Table 1).

**Table 1:** Goals and objectives of the academic elective.

Goals	Objectives
Equip residents to “teach on the fly” when busy and teach in difficult scenarios, give residents a framework for assessing the learner, and focus on specific skills such as physical exam and chalk talk teaching	Learn and implement Resident as Teachers skills
Provide opportunities for residents to implement teaching tools in inpatient and outpatient settings, and to have these teachings observed by attending physicians	Join a medicine wards team and pediatric wards team as their teaching consultant
Improve resident ability to give effective feedback and increase resident comfort with awkward feedback scenarios	Practice giving difficult feedback using feedback scripts
Give residents confidence in building and executing a formal teaching lecture	Develop and teach a morning report and MS3 didactic session
Provide opportunities for residents to apply evidence-based skills in real time, clinically integrated settings and teach their knowledge	Learn and implement EBCP tools
Help residents to enhance their evidence-based clinical practice skills	Join a medicine wards team and pediatric wards team as their evidence consultant
Give residents tools to critically analyze and apply primary literature to their practice	Critically analyze Grand Rounds presentations
Provide resident wellness and to equip residents with simple methods to bring wellness to their teams	Learn and implement Wellness tools. Join a medicine wards team and pediatric wards team as their wellness consultant
Equip residents in anti-racism knowledge and skills	Engage and participate in difficult discussions regarding race and medicine
To develop a program-wide repository for chalk talks and teaching tools	Create 3 chalk talks/teaching handouts and upload them to the program-wide repository

**Figure 1:**
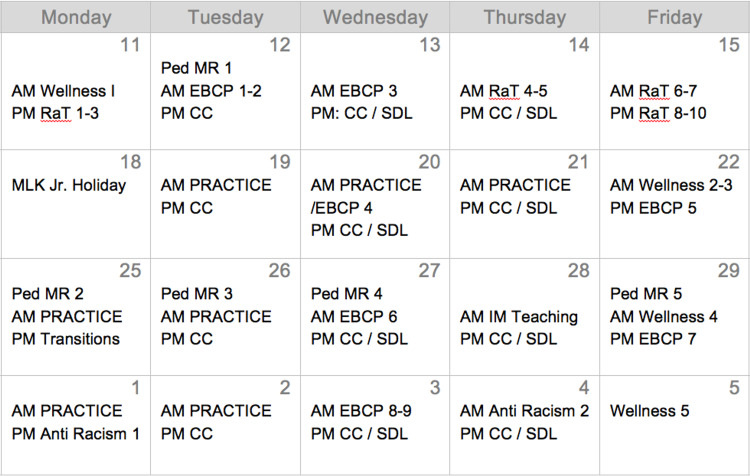
Bird’s eye schedule of the academic elective. The schedule ran from January 11, 2021 to February 5 2021. Abbreviations are as follows: RaT = residents as teachers; EBCP = evidence-based clinical practice; CC = continuity clinic; SDL = self-directed learning including developing a morning report, contributing to teaching talk repository, and completing reading for future sessions; PRACTICE = consultant on outpatient, subspecialty, or inpatient practice, implementing EBCP, RaT, wellness, and feedback skills.

During the summer and fall of 2020, speakers were identified and invited to give a teaching session during the elective. Based on speaker availability and resident continuity clinic, a schedule was developed (Figure 1). One resident had matched into a fellowship in transitional medicine and asked for time to give a presentation on this topic, which was also scheduled. Morning sessions were scheduled from 8:30 AM to 12 PM and afternoon sessions were scheduled from 1 PM to 5 PM. Residents were expected to attend pediatric morning reports from 8 AM to 8:30 AM as well as either pediatric or internal medicine noon conference educational sessions virtually. Residents were also still scheduled for their continuity clinic two afternoons per week.

Residents as Teachers

For the RaT component, several curricula were reviewed including Alliance of Academic Internal Medicine lectures from the MedEd portal, Mount Sinai Resident Teaching Development program, Society of Teachers of Family Medicine lectures, the University of Virginia Resident as Teacher, and NEJM Resident as Teacher [25-29]. All of these were publicly available resources. These resources were compiled into a document and analyzed. The following topics were chosen: general principles of resident as a teacher, setting goals, giving and receiving feedback, professionalism, teaching from oral case presentations, teaching from documentation, teaching from the physical exam, clinical reasoning, giving effective presentations, leading rounds, and giving effective chalk talks. Nine faculty members (four from the department of pediatrics, four from the department of internal medicine, and one with appointments in both departments of internal medicine and pediatrics) were identified as experts and agreed to give lectures in these areas.

Evidence-based clinical practice

The EBCP portion was modified from an existing elective offered at the University of Utah School of Medicine for both fourth-year medical students and upper-level residents. Lectures included the following topics: acquiring evidence, appraisal, prognosis, systematic reviews, clinical decision tools, diagnostic studies, harm studies, meta-analysis, qualitative research, and regression. “Users’ Guides to the Medical Literature: A Manual for Evidence-Based Clinical Practice” was used as a textbook [30]. Nine faculty members (two from the department of pediatrics, one from the department of internal medicine, four with appointments in both departments of internal medicine and pediatrics, and two librarians) were identified as experts and agreed to give lectures in these areas. There was a separate two-week elective offered to medical students which overlapped and interfaced with the resident academic elective. The residents were assigned one EBCP topic to teach to the medical students.

Wellness

Wellness sessions were included weekly and were based on a newly implemented Graduate Medical Education (GME) wellness elective as well as the resident survey. The topics covered included mindfulness and meditation, medical narrative writing, determination of values, and incorporating pause practices into wards. These sessions were led by three faculty members, as well as the Wellness Director of the Office of Graduate Medical Education. Wellness sessions were grounded in core concepts of psychological safety, with opportunities to discuss causes and signs of burnout, use specific reflection and mindfulness practices to cultivate resilience, and practice self-compassion. Guided meditation and journaling prompts were integrated into each session. Faculty leaders demonstrated a commitment to modeling vulnerability and normalizing experiences of impostor syndrome and emotional exhaustion. Residents were invited to share experiences of burnout and stories of resilience in ways that fostered solidarity and community. Residents were asked to commit to three goals for self-care and self-compassion during the month. In addition, the residents were given one day away from didactic teaching to engage in a group exercise outside of the hospital.

Anti-racism

Anti-racism sessions included sessions on white identity, microaggressions, and implicit bias, with a focus on psychological safety throughout these discussions. These sessions were led by three faculty members as well as a staff member from the Office of Health Equity, Diversity, and Inclusion and built on prior sessions that had been completed during previous Med-Peds didactic half-day sessions. During the sessions, participants received training in anti-racist concepts, took an implicit bias test, practiced responding to a simulated microaggression with tools that promote upstanding, and reflected on behaviors and practices that foster inclusivity. Participants debriefed about prior witnessed/experienced microaggressions and were encouraged to report these examples with faculty support.

Core practice

The residents were given several avenues to practice and implement the material learned during the elective. Six mornings were set aside for “Core Practice” sessions, where the resident joined an inpatient pediatric team, inpatient internal medicine team, or an outpatient Med-Peds provider and used clinical encounters to 1) ask clinical questions and critically identify and appraise the evidence, 2) craft and deliver short “on-the-fly” teaching moments or chalk talks, and 3) lead wellness exercises. Residents kept a log of the clinical questions, teaching topics, and wellness activities they performed during these sessions. In addition, residents were asked to create and upload three chalk talks onto a common online repository, which served as a “deliverable” for future teaching opportunities. Lastly, residents were assigned to present at a pediatric morning report session on a topic of their choosing as well as give a didactic lecture for third-year medical students currently on their internal medicine clerkship.

Analysis

Lectures and experiences were evaluated by each participant using a Likert scale from 1 to 7, with 1 defined as “not valuable at all” and 7 defined as “extremely valuable.” The mean and 95% confidence intervals were calculated and reported.

## Results

The elective was conducted from January 11, 2021 to February 7, 2021. Due to COVID-19 restrictions, most of the lectures were presented over Zoom, with only four lectures in person. The Core Practice was all done in clinical settings and in person. For one week, one resident was pulled to cover the COVID ICU and thus unable to participate in the material. Otherwise, there were no changes to the original plan and every lecture was given successfully.

**Figure 2:**
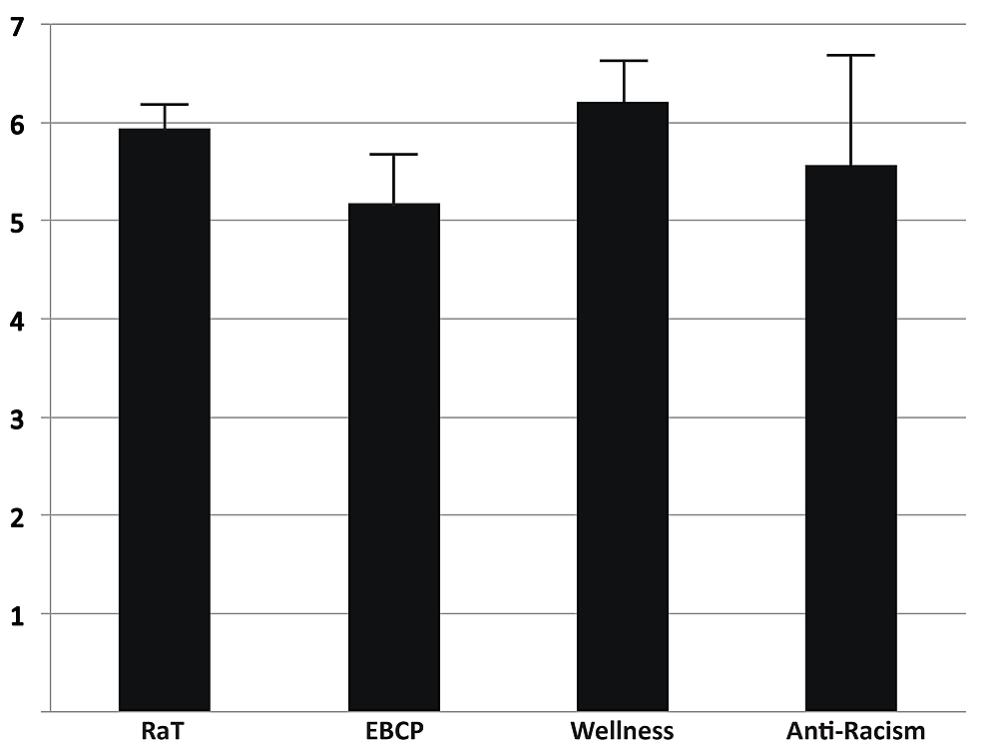
Student-rated value of each lecture topic. Each lecture was categorized into one of the following four topics: residents as teachers (RaT), evidence-based clinical practice (EBCP), wellness, and anti-racism (displayed on x-axis). Likert scale rating for each student was obtained for each lecture (1 = not valuable at all, 7 = extremely valuable) and these scores were averaged for each category. Bar graph represents mean with whisker above representing upper 95% confidence interval.

Lectures were categorized into four topics: RaT (10 lectures), EBCP (nine lectures), wellness (four lectures), and anti-racism (two lectures). Each lecture was rated by each resident (n = 4-5 per lecture) by a Likert scale according to the value-added to their education. The average score for each category was compiled and reported (Figure 2). Wellness lectures were found to have the highest value-added material (6.20 ± 0.41, n = 18), followed by RaT lectures (5.93 ± 0.25, n = 48), anti-racism (5.57 ± 1.11, n = 9), and EBCP (5.18 ± 0.50, n = 43). The highest yield topics were determined as topics that only scored 6 or 7 by every resident. The highest yield individual topics were as follows: Chalk Talks, mean = 7 (RaT), Values, mean = 6.8 (wellness), Giving Feedback, mean = 6.5 (RaT), Oral Presentations, mean = 6.5 (RaT), Reviews and Guidelines, mean = 6.4 (EBCP), Mindfulness, mean = 6.4 (wellness), and Implicit Bias and Microaggressions, mean = 6.4 (anti-racism).

Qualitative comments were also obtained for each lecture (Table 2). Several topics were highlighted as helpful and not encountered in training previously, including teaching from documentation and giving a formal presentation. Only one lecture was noted to be difficult in the online environment. Comments were also obtained regarding the Core Practice components of the elective, which were generally felt to be helpful, particularly the sessions where the resident served as a real-time teaching or evidence consultant during inpatient rounds.

**Table 2:** A sample of qualitative comments of lectures and practice sessions. Some comments were edited in a minor way for clarity or brevity.

Lecture	Comment
RaT	
Professionalism	… an important subject to revisit at every stage of medical training.
Rounds	… made me feel more empowered to take a leadership role on rounds.
Presentations	We don’t get enough formal education on presentations … neglected in our training
Documentation	We never learn how to critique and give feedback on notes
Feedback	Feedback scripts were new information for me
EBCP	
Prognosis	Would have been more helpful to have the article ahead of time
Ask/Aquire	Challenging over Zoom, practicing PICO was helpful
Treatment	Packed with information
Wellness	
Pause Practices	I can use this when on wards and teams
Anti-Racism	
Implicit Bias	Thoroughly appreciated this insightful and thoughtful discussion with experts and peers
Practice	
MS3 Teaching	Interesting to understand how certain topics may be taught with a script
Practice	Great real-life practice! Although somewhat difficult in actual implementation

## Discussion

We were able to create and execute an academic elective with dedicated time for upper-level residents to learn and explore the four domains of resident teaching, EBCP, wellness, and anti-racism work. The material was generally highly rated and was delivered by 25 faculty members across four different departments. Notably, all lectures were given on a volunteer basis and none were reimbursed for their time. The lectures were conducted in a mostly online environment due to COVID-19. In addition, residents contributed to an online repository that currently has material for 25 chalk talks as well as the slides from the elective sessions.

Given the favorable response to this elective, we have scheduled our rising second- and third-year residents to receive this elective next year and will use the evaluations we received to modify our course to make it more effective. We hope to increase the number of experiential sessions, especially in regards to chalk talks and wellness. We would also like to have an increased amount of education in anti-racism, particularly on how structural racism impacts healthcare locally. We are also hoping to be able to have more sessions in person, although this will need to be evaluated closer to the elective dates next year.

In addition, we have been approached by the University of Utah Internal Medicine program as well as the Pediatrics program to collaborate on offering a version of this elective to categorical IM and categorical Pediatric residents. We are currently working with program directors from each of these categorical programs to create two-week versions of the elective for categorical residents embedded within the four-week elective for Med-Peds residents.

Strengths and limitations

Because this elective was four weeks, we were able to address four different domains of resident learning and provide practice time for these domains. Indeed, a unique piece of this elective was the core practice portion, which allowed residents to hone their teaching and EBCP skills in real-time, without the responsibilities of clinical duties. We were also able to combine EBCP components with the concurrent fourth-year medical student elective, which created opportunities for residents to teach concepts to the medical students. Lastly, we were able to recruit a diverse, robust set of faculty to facilitate these sessions. In terms of limitations, our survey data were limited by our small sample size, but as this was a pilot year, we opted to limit the rotation to Med-Peds residents. In addition, residents likely received other education in these domains from sessions offered through the categorical Pediatric and Internal Medicine programs at our institution, which may confound our data. To mitigate this, we used surveys assessing each lecture for its value rather than surveying other metrics such as resident confidence or proficiency in a given domain. Another limitation is that our elective occurred during the COVID-19 pandemic, and many sessions were moved online. Fortunately, the core practice component of the elective was still able to be in person.

## Conclusions

We were able to create and execute an academic elective with dedicated time for upper-level residents to develop and utilize valuable skills in teaching, evidence appraisal, wellness, and anti-racism. Future work will focus on refining the curriculum based on resident evaluations and expanding this elective to the Internal Medicine and Pediatrics categorical programs at our institution.
